# A social norms approach intervention to address misperceptions of anti-vaccine conspiracy beliefs amongst UK parents

**DOI:** 10.1371/journal.pone.0258985

**Published:** 2021-11-12

**Authors:** Darel Cookson, Daniel Jolley, Robert C. Dempsey, Rachel Povey

**Affiliations:** 1 Department of Psychology, School of Social Sciences, Nottingham Trent University, Nottingham, United Kingdom; 2 Department of Psychology, Northumbria University, Newcastle upon Tyne, United Kingdom; 3 Department of Psychology, Faculty of Health, Psychology and Social Care, Manchester Metropolitan University, Manchester, United Kingdom; 4 The Staffordshire Centre for Psychological Research, School of Life Sciences and Education, Staffordshire University, Stoke-on-Trent, United Kingdom; University of Haifa, ISRAEL

## Abstract

Anti-vaccine conspiracy beliefs among parents can reduce vaccination intentions. Parents’ beliefs in anti-vaccine conspiracy theories are also related to their perceptions of other parents’ conspiracy beliefs. Further, research has shown that parents hold misperceptions of anti-vaccine conspiracy belief norms: UK parents over-estimate the anti-vaccine conspiracy beliefs of other parents. The present study tested the effectiveness of a Social Norms Approach intervention, which corrects misperceptions using normative feedback, to reduce UK parents’ anti-vaccine conspiracy beliefs and increase vaccination intentions. At baseline, 202 UK parents of young children reported their personal belief in anti-vaccine conspiracy theories, future intentions to vaccinate, and their perceptions of other UK parents’ beliefs and intentions. Participants were then randomly assigned to a normative feedback condition (*n* = 89) or an assessment-only control condition (*n* = 113). The normative feedback compared participants’ personal anti-vaccine conspiracy beliefs and perceptions of other UK parents’ beliefs with *actual* normative belief levels. Parents receiving the normative feedback showed significantly reduced personal belief in anti-vaccine conspiracy beliefs at immediate post-test. As hypothesised, changes in normative perceptions of anti-vaccine conspiracy beliefs mediated the effect of the intervention. The intervention, did not directly increase vaccination intentions, however mediation analysis showed that the normative feedback increased perceptions of other parents’ vaccination intentions, which in turn increased personal vaccination intentions. No significant effects remained after a six-week follow-up. The current research demonstrates the potential utility of Social Norms Approach interventions for correcting misperceptions and reducing anti-vaccine conspiracy beliefs among UK parents. Further research could explore utilising a top-up intervention to maintain the efficacy.

## 1. Introduction

Social norms are informal, unwritten expectations about appropriate social behaviour, outlining what is acceptable and not in particular contexts, and are important determinants of attitudes and behaviours [1–3]. Two types of social norms are commonly implicated in health behaviours: injunctive norms, which refer to one’s perception of what others approve or disapprove [1], and descriptive norms, which refer to one’s perception of how prevalent an attitude or behaviour is [4]. Perceived social norms are an important predictor of health behaviours [5]. Specifically, perceived norms of anti-vaccine conspiracy beliefs predict personal belief in anti-vaccine conspiracy theories among parents [6]. Anti-vaccine conspiracy beliefs among parents can reduce intentions to vaccinate, which is problematic as UK childhood vaccination rates are below target [7]. Social Norms Approach (SNA) interventions have been successfully used to improve health attitudes and behaviours by challenging commonly held misperceptions or misestimates of actual norms [8]. Thus, the current study aims to utilise the SNA intervention to reduce belief in anti-vaccine conspiracy theories and increase vaccination intentions among UK parents.

The Social Norms Approach (SNA) begins with the premise that individuals are influenced by the beliefs and behaviours of others and often make misperceptions about how much others engage in certain behaviours [8, 9]. For example, people tend to over-estimate how much others engage in negative health behaviours, such as drinking alcohol (e.g., [10]), smoking tobacco (e.g., [11, 12]), and unhealthy snacking [13]. As social norms can provide an expectation about appropriate social behaviour [1, 2], individuals may be driven to match what they perceive to be the social norm [14]. An important consequence of such social norm misperceptions is the potential engagement in unhealthy behaviours due to a false belief that such behaviours are commonplace amongst peers [9, 15]. The SNA works by challenging these misperceptions of the belief and behaviours of others, reducing the perceived social pressure to engage in a problem behaviour, to promote healthier personal behaviours [8]. SNA interventions are often delivered online using computerised normative feedback to explicitly compare a) personal beliefs and behaviours to b) the perceived norms of peers and to c) the actual norms of a certain belief or behaviour (e.g., [16]). Therefore, this feedback explicitly demonstrates existing misperceptions in people’s perceptions of peer norms and highlights their norm deviant behaviours [8]. For example, normative feedback has often been used to reduce college student drinking [16], where correcting misperceptions has been shown to reduce perceived drinking norms and, in turn, reduce personal drinking. The SNA is also gaining traction in other areas, for example, increasing sun-protective behaviours [17], increasing cancer screening intentions [18], and reducing problematic gambling [19, 20]. The objective of the present study is to reduce anti-vaccine conspiracy beliefs and increase vaccination intentions using an SNA intervention.

Conspiracy theories are alternative explanations for events that implicate secretive and powerful groups in covering up information to suit their interests [21]. Examples of conspiracy theories include the idea that climate change is a hoax [22, 23], COVID-19 is caused by electromagnetic waves transmitted by 5G technology [24], and that vaccines are dangerous, but this is covered up to maintain profits [25]. Belief in conspiracy theories can have potentially detrimental health consequences [26]. Of central interest to this research is that exposure to anti-vaccine conspiracy theories directly increases belief in them, which reduces intentions to vaccinate [25, 27]. Specifically, Hornsey et al. [27] found that anti-vaccination attitudes were highest among those who were higher in conspiratorial thinking, and Jolley and Douglas [25] showed that belief in anti-vaccine conspiracy theories was a causal factor in reduced vaccination intentions.

Vaccine hesitancy is defined by the World Health Organisation (WHO) as “the reluctance or refusal to vaccinate despite the availability of vaccines” and reduces vaccination intentions and uptake (e.g., [28]). The WHO listed vaccine hesitancy as a top ten threat to global health in 2019 [29], and in the UK specifically, childhood vaccination rates have been steadily decreasing since 2013, with a slight increase from 2019–2020 [7]. As a result, governments around the world have debated mandatory vaccinations, for example, Australia, France, and Italy have restricted school access for children who have not received their scheduled vaccinations [30]. Highlighting the current urgency to address the challenges of vaccination access and uptake globally, The World Health Assembly has endorsed a new global Immunization Agenda 2030 (IA2030), aiming to maximise the potential of vaccines worldwide [31]. Since vaccines have been used in the UK, several childhood diseases which could be fatal (e.g., smallpox and polio) have been eradicated [32]. However, some diseases, like measles and mumps are starting to appear again, where in the UK cases have almost doubled in recent years [32]. Measles outbreaks continue to occur in Europe, resultant of suboptimal vaccine uptake [33, 34]. For example, a large measles outbreak in South Wales in 2012/2013 was attributed to low uptake of the measles-mumps-rubella (MMR) vaccine, where belief in conspiracy theories around that specific vaccine was reported by parents as an influence on their decision [35]. Considering the ongoing COVID-19 pandemic, where at least seven COVID-19 vaccines are being rolled out across the world [36], it is imperative to understand how to increase vaccination intentions to promote their uptake [37]. Research is already showing that anti-vaccine conspiracy beliefs are associated with the rejection of COVID-19 vaccines (e.g., [38]).

Previous research into interventions to increase vaccination intentions has been inconsistent [39]. For example, two systematic reviews of interventional studies, aiming to address parental vaccine hesitancy and refusal, could not identify a specific form of interventional approach to reduce parental vaccine hesitancy and refusal [39, 40]. Although research has consistently linked anti-vaccine conspiracy ideation with vaccine hesitancy [25, 27, 41], little research has focussed on addressing these beliefs as a mechanism of increasing vaccination intentions. Jolley and Douglas [42] employed an inoculation technique and demonstrated that exposure to anti-conspiracy arguments before exposure to anti-vaccine conspiracy theories could reduce belief. However, this intervention was not successful when the anti-conspiracy arguments were presented after the anti-vaccine conspiracy theories. Whilst inoculation approaches could be useful for reducing anti-vaccine beliefs, counterarguments may not be effective when conspiracy beliefs are already established. Therefore, there is a need to develop and test novel interventions to address anti-vaccine conspiracy beliefs and increase vaccination intentions in parents.

The current study aims to test the utility of a brief online normative feedback SNA intervention to reduce anti-vaccine conspiracy theories and increase vaccination intentions among UK parents of young children. Cookson et al. [6] found that UK parents’ anti-vaccine conspiracy beliefs are strongly associated with perceived norms of other parents’ anti-vaccine conspiracy beliefs and that UK parents over-estimated the extent that other parents endorsed these conspiracy theories. Therefore, an SNA intervention using normative feedback to correct these misperceptions could reduce anti-vaccine conspiracy beliefs and, consequently, increase vaccination intentions. Previous research has used different lengths of time for a follow-up to the intervention to measure maintained efficacy, and while four weeks had been considered short [17], the computer-delivered normative feedback employed in the current research is brief and as such, a six-week follow-up will be used. The current study randomly allocated participants to either the SNA condition, which uses normative feedback to correct misperceptions of anti-vaccine conspiracy beliefs and vaccination intentions, or to an assessment only control condition. The key hypotheses are that: 1) participants in the intervention condition will have a reduced belief in anti-vaccine conspiracy theories from baseline to immediate post-test and at the six-week follow-up, compared to the control condition; 2) participants in the intervention condition will have an increased intention to vaccinate from baseline, to immediate post-test and six-week follow up, compared to the control condition; 3) The effect of the intervention on belief in anti-vaccine conspiracy theories will be mediated by a change in perceived norms of other parents’ anti-vaccine conspiracy beliefs, and 4) The effect of the intervention on vaccination intentions will be mediated by the change in the perceived norms of other parents’ vaccination intentions. This study was pre-registered (https://osf.io/cdp53) and the materials and anonymous data can be accessed here: https://osf.io/rhb5p/

## 2. Method

### 2.1. Participants

**Fig 1:**
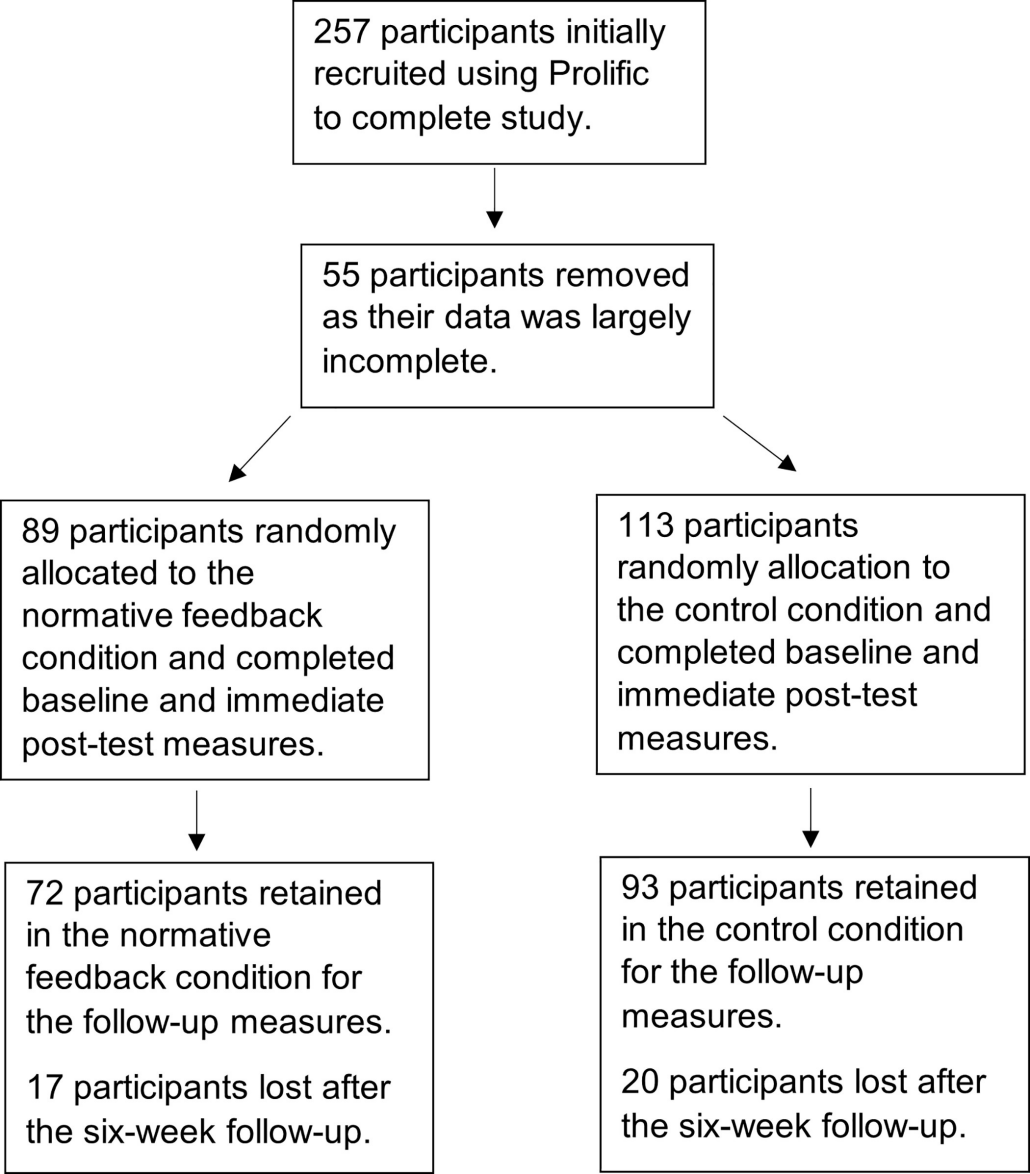
Flow-chart showing the number of participants in each stage of the study.

Fig 1 depicts the number of participants at each stage of the study. There have been no previous studies using an SNA based intervention to reduce belief in anti-vaccine conspiracy theories or to increase vaccination intentions, therefore, there were no clear expectations of effect size. Other studies using SNA interventions to improve health behaviours tend to find small to medium effect sizes (e.g., [17]), and a previous intervention aiming to increase vaccination intentions using anti-conspiracy counterarguments found a medium effect size [42]. For the main mixed factorial ANOVA analysis, a power analysis using GPower [43] was conducted and showed that to detect a small-medium effect using Cohen’s d (*d* = .35), a sample of *n* = 174 participants would be required for 80% power. Similarly, a sample size of at least 148 participants is recommended for mediation analysis expecting a small-medium effect [44]. Therefore, anticipating incomplete data and potential drop-out, 257 participants were recruited using *Prolific*, an online recruitment platform where volunteers can register for studies in return for small monetary rewards.

Screening criteria were applied via *Prolific* to ensure that all participants were British and had a high approval rating on the recruitment platform, meaning that they had a reputation for completing surveys satisfactorily. To ensure participants were suitable for the intervention’s focus on vaccines, screening criteria on Prolific were also used to ensure that participants had a child aged 5 years or younger and had previously stated when signing up to the platform that they did not ‘totally agree’ that scheduled vaccines were safe for children. Several incomplete responses were removed from the dataset (*n* = 55). The remaining participants (*N* = 202; 160 females, 41 males, 1 trans/other, *M* = 34.16 years, *SD* = 5.39) were included in the analysis; *n* = 89 in the experimental condition and *n* = 113 in the control condition.

### 2.2 Design

The study employed a 2*3 (intervention condition by time) mixed experimental design. After completing baseline measures, participants were randomly allocated, using the Qualtrics block randomiser, to the experimental condition, where they would receive SNA normative feedback, or the assessment only control condition. The measures were completed at three time points: baseline, immediately after the intervention (for the control condition, this was immediately after a 60-second delay), and then a six-week follow-up, where participants were contacted again via *Prolific*. There were four dependent variables measured at each of the three-time points: 1) personal beliefs in anti-vaccine conspiracy theories; 2) perceived belief of other parents in anti-vaccine conspiracy theories; 3) vaccination intentions; and 4) perceived vaccination intentions of other parents. Demographic variables including participant’s own gender, age, age of youngest child and education level were also measured.

### 2.3 Materials and procedure

Ethical approval was first gained from the relevant university ethics panel. The study consisted of three phases, each completed online using Qualtrics, an online software tool used to build questionnaires and experiments. The first phase was baseline assessment, followed immediately by the intervention (or control) and immediate post-test measures (August 2020), and finally the six-week follow up (September 2020).

Once the study was accessed, participants were presented with an information page, followed by a consent form. First, participants were asked to complete the demographic questions followed by a one-item scale to measure their general belief in conspiracy theories [45]. Then, baseline measurements of the dependent variables were then taken. Personal belief in anti-vaccine conspiracy theories was measured using the *Belief in Anti-Vaccine Conspiracy Theories Scale*, adapted from [25]. There were 10 statements for participants to complete (e.g., “Misrepresentation of the efficacy of childhood vaccines is motivated by profit”, 1 = strongly disagree, 7 = strongly agree, α = .96). Participants completed this scale for a second time, where it was adapted to measure the perceived beliefs of the “*majority of other UK parents”* (α = .95). Participants’ intentions to vaccinate was then measured using a scenario used widely in previous research [25, 42, 46, 47]. In this scenario, participants are asked to imagine that they were the parent of an infant named Sophie, aged 8 months, and that their doctor had provided them with information regarding the (fictitious) disease ‘dysomeria’, which may lead to serious consequences with symptoms such as fever and vomiting. After reading the scenario, participants indicated their intention to have Sophie vaccinated (“If you had the opportunity to vaccinate your child (Sophie, aged 8 months) against ‘dysomeria’ next week, what would you decide”) on a scale of 1–7 (1 = definitely not vaccinate to 7 = definitely vaccinate). Then participants were asked this question again, but to answer with their perception of how other British parents would respond.

Participants were then randomly allocated to the SNA normative feedback condition or to the assessment only control condition. Participants in the normative feedback condition received a page of feedback (S1 File) which included personal feedback comparing a) their belief in anti-vaccine conspiracy theories; b) their estimation of how much other UK parents believed in them; and c) the *actual* belief of other parents in anti-vaccine conspiracy theories. The ‘actual’ belief of UK parents in anti-vaccine conspiracy theories was taken as an average from previous research conducted by the authors into UK parents’ belief in anti-vaccine conspiracy theories (e.g., [6]). In line with the Social Norms Approach (e.g., [8]), these three norms could be compared to demonstrate that misperceptions of the beliefs and behaviours of others are common and that belief in anti-vaccine conspiracy theories is very low amongst other UK parents. The feedback also indicated that “The development of vaccines is one of the most important advances in the history of medicine” and that “The overwhelming majority of UK parents choose to vaccinate their children”. Participants were presented with this page for 60 seconds before they could proceed to the post-intervention assessment. Participants who were allocated to the control condition did not receive any feedback. Instead, they were instructed to click through some waiting screens for the same amount of time (60 seconds) before moving on to the post-intervention assessment.

Immediately after the intervention, all the dependent variables were measured again; the *Belief in Anti-Vaccine Conspiracy Theories Scale* [25] from participants own point of view (Time 2 *α* = .97) and from the perspective of other UK parents (Time 2 *α* = .97); and the intention to vaccinate from their own point of view and from the perspective of other UK parents’ intentions [47]. Participants were then invited to complete a follow-up questionnaire after six weeks, given a shortened debrief, and thanked for their time.

At the six-week follow up, all four dependent variables were measured again (Belief in Anti-Vaccine Conspiracy Theories Scale [25] from participants own point of view (Time 3 α = .96) and from the perspective of other UK parents (Time 3 α = .96); and the intention to vaccinate from their own point of view and from the perspective of other UK parents’ intentions [47]. Once these measures were completed, participants were thanked for their time and fully debriefed.

## 3. Results

### 3.1 Baseline equivalence of conditions

Baseline equivalence was measured using independent samples t-tests or chi-square models to ensure that the normative feedback condition and control condition were matched across key variables. Table 1 highlights no significant differences across baseline variables between the normative feedback condition and the control condition. There were also no significant differences between conditions in gender of participants (*χ^2^* (2, *N* = 202) = 3.32, *p* = .191). The data were also checked for parametric assumptions. The perceived belief of other parents in anti-vaccine conspiracy theories was positively skewed; thus, this variable was transformed at each timepoint using the square root transformation. Participants’ intentions to vaccinate and their perceived intention of other parents to vaccinate were both negatively skewed, and as such, these variables were transformed using the square transformation at each timepoint. The transformations addressed the skew.

**Table 1:** Means, standard deviations and equivalence tests between the normative feedback and control conditions of baseline measures.

	Normative Feedback Condition	Control Condition	
	*Mean (SD)*	*Mean (SD)*	*t (df)*
Age	33.91 (5.97)	34.35 (4.91)	-.57 (169.13)
Age of youngest child	2.46 (1.32)	2.24 (1.36)	1.13 (200)
Education level	5.43 (1.06)	5.39 (1.08)	.25 (190.32)
General belief in conspiracy theories	3.87 (1.02)	3.81 (1.16)	.38 (200)
Baseline belief in anti-vaccine conspiracy theories	2.85 (1.40)	2.72 (1.36)	.67 (200)
Baseline perceived belief of other parents	3.23 (1.15)	3.16 (1.29)	.42 (200)
Baseline intentions to vaccinate	5.83 (1.42)	5.87 (1.36)	-.18 (200)
Baseline perceived intentions of other parents	5.49 (1.11)	5.43 (1.19)	.37 (200)

### 3.2 Baseline support for SNA

We first conducted regression analysis among variables at baseline to provide additional justification for an SNA-based intervention. This analysis largely replicated the findings of Cookson et al. [6]. Consistent with the rationale, perceived norms of other UK parents’ belief in anti-vaccine conspiracy theories significantly positively predicted personal anti-vaccine conspiracy belief, *F*(4, 197) = 23.67, *R^2^ * =  .32, *p* < .001. Similarly, a paired samples t-test, comparing participants’ anti-vaccine conspiracy beliefs and their perceptions of other parents’ beliefs, showed that participants significantly over-estimated the conspiratorial beliefs of others, *t*(201) = -4.56, *p* < .001, *d* = 0.32. The same pattern of results was found with participants’ intentions to vaccinate. At baseline, perceived norms of vaccination intentions of other UK parents significantly positively predicted personal vaccination intentions of UK parents *F*(2, 199) = 21.02, *R^2^ * =  .17, *p* < .001. A paired samples t-test, comparing participants’ vaccination intentions and their perceptions of other parents’ intentions, demonstrated that participants significantly under-estimated the vaccination intentions of other UK parents, *t*(201) = 5.14, *p* < .001, *d* = 0.31. Thus, baseline analysis confirmed that anti-vaccine conspiracy beliefs and vaccination intentions meet the key criteria for an SNA based intervention (misperceptions of social norms and misperceptions being predictive of personal behaviours/intentions; e.g., Dempsey et al. [8].

### 3.3 Attrition

A total of 165 from the 202 participants who agreed to be contacted completed the follow up questionnaire six weeks later (18% drop-out rate; normative feedback condition: *n* = 72, control condition: *n* = 93). Rates of attrition did not differ between conditions at follow-up, *χ^2^(*1, *N* = 202) = .07, *p* = .798. There were no differences in gender, *χ^2^(*2, *N* = 202) = .29, *p* = .866; age, *z*(2930.5) = -.38, *p* = .704; education level, *z*(3001) = -.17, *p* = .867; general conspiracy beliefs, *z*(3018.5) = -.11, *p* = .915; baseline beliefs in anti-vaccine conspiracy theories, *z*(2829) = -.70, *p* = .486; or baseline vaccination intentions, *z*(2848.5) = -.67, *p* = .503 between participants who completed the follow up measures and participants who did not. Thus, the following analyses were conducted with the 165 retained participants.

### 3.4 Hypothesis 1: Impact of the intervention on personal belief in anti-vaccine conspiracy theories

Descriptive statistics are presented in Table 2. The impact of the intervention on personal beliefs in anti-vaccine conspiracy theories was investigated using a mixed factorial ANCOVA. Education level was entered as a covariate, as education level was related to personal beliefs in anti-vaccine conspiracy theories. Mauchly’s test of sphericity showed that the assumption of sphericity was violated (*p* < .001), therefore the Greenhouse Geisser correction was used. No main effects were significant (see S1 Table). There was a significant interaction between time and condition on belief in anti-vaccine conspiracy theories, indicating the effectiveness of the intervention, *F*(1.56, 253.27) = 4.73, *p* = .016, *η*_*p*_^*2*^ = .03. Pairwise comparisons showed no significant differences in anti-vaccine conspiracy belief across the three-time points in the control condition. However, in the normative feedback condition, anti-vaccine conspiracy beliefs significantly decreased from baseline (*M* = 2.81, *SD* = 1.41) to immediate post-test (*M* = 2.50, *SD* = 1.42) (*p* < .001). However, belief significantly increased again from immediate post-test to the six-week follow up (*M* = 2.79, *SD* = 1.27) (*p* = .020). There was no difference in belief in anti-vaccine conspiracy theories from baseline to the six-week follow up (p = 1).

**Table 2:** Means and standard deviations of each dependent variable for each condition across the three time points.

	Normative Feedback Condition			Control Condition		
	Baseline	Immediate post-test	Six-week follow-up	Baseline	Immediate post-test	Six-week follow-up
	*Mean (SD)*	*Mean (SD)*	*Mean (SD)*	*Mean (SD)*	*Mean (SD)*	*Mean (SD)*
Belief in anti-vaccine conspiracy theories	2.81 (1.41)	2.50 (1.42)	2.79 (1.27)	2.69 (1.32)	2.71 (1.41)	2.71 (1.42)
Perceived belief of other parents	3.19 (1.13)	2.53 (1.23)	2.97 (1.18)	3.27 (1.29)	3.14 (1.17)	3.09 (1.22)
Intentions to vaccinate	5.81 (1.51)	5.88 (1.55)	5.94 (1.35)	5.96 (1.23)	5.90 (1.29)	6.03 (1.32)
Perceived intentions of other parents	5.43 (1.16)	5.78 (1.15)	5.68 (1.09)	5.42 (1.07)	5.26 (1.23)	5.60 (1.03)

### 3.5 Hypothesis 2: Impact of the intervention on personal vaccination intentions

The impact of the intervention on personal vaccination intentions was then examined using a mixed factorial ANCOVA (S2 Table). As in the previous analysis, education level was entered as a covariate. Mauchly’s test of sphericity was violated (*p* < .001) and the Greenhouse Geisser correction was used. The ANCOVA showed that there were no significant main effects (S2 Table), and there was no significant interaction between time point and experimental condition, *F*(1.37, 222.57) = .55, *p* = .515. Therefore, there was no significant effect of the intervention on increasing vaccination intentions across the three time points. Overall, the SNA type intervention reduced UK parents’ belief in anti-vaccine conspiracy theories at immediate post-test but did not significantly increase vaccination intentions.

### 3.6 Hypothesis 3: The mediating role of *perceived norm change* in the impact of the SNA intervention on belief in anti-vaccine conspiracy theories

**Fig 2:**
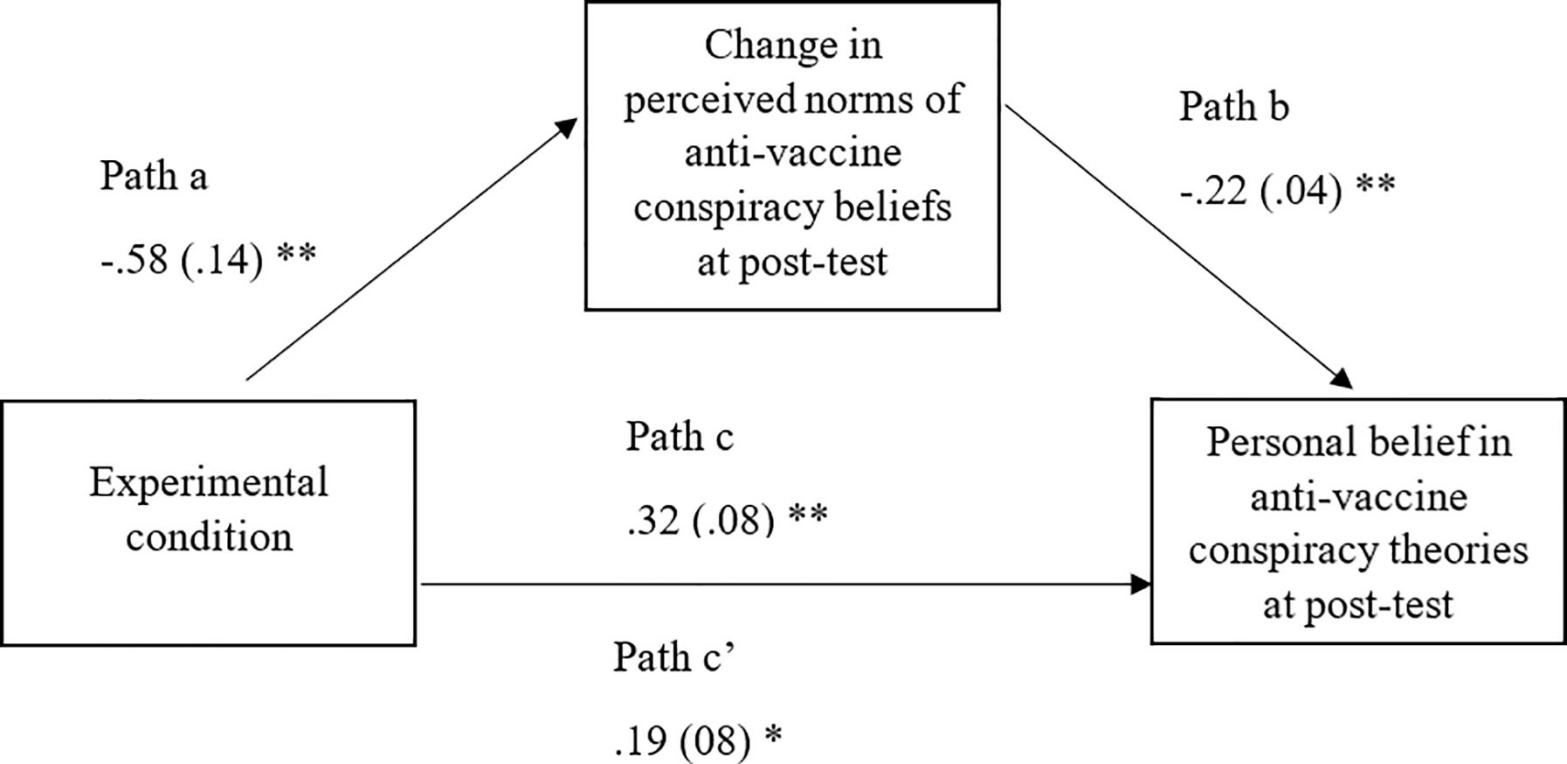
Model 4 showing mediation of the experimental condition reducing anti-vaccine conspiracy beliefs through reduced perceived norms of anti-vaccine conspiracy beliefs. * = p < .05, ** = p < .001.

Mediation analysis was employed to examine the mechanism through which the normative feedback reduced belief in anti-vaccine conspiracy theories at immediate post-test. Specifically, this analysis tested the hypothesis that the change in perceived norms of other parents’ anti-vaccine conspiracy beliefs from baseline to immediate post-test mediates the influence of the SNA normative feedback on personal anti-vaccine conspiracy beliefs. The change in perceived norms of other parents’ anti-vaccine conspiracy beliefs variable was calculated by subtracting participants’ perceived beliefs after the intervention from their baseline perceptions. Therefore, a positive number indicates that perceptions of other parents’ beliefs in anti-vaccine conspiracy theories have decreased. The mediation analysis was conducted using Model 4 the PROCESS macro for SPSS, with 5000 bootstrapped samples [48]. Baseline belief in anti-vaccine conspiracy theories and perceived norms of anti-vaccine conspiracy theories were included in the model as covariates. The analysis supported the hypothesis (Fig 2). The normative feedback condition significantly reduced perceived norms of conspiracy belief at immediate post-test, *b* = -.58, *SE* = .14, *t*(161) = -4.22, *p* < .001, CI [-.85, -.31]. Similarly, a change in perceived conspiracy belief at immediate post-test significantly predicted personal anti-vaccine conspiracy beliefs immediately after the intervention, *b* = -.22, *SE* = .04, *t*(160) = -5.14, *p* < .001, CI [-.31, -.14]. The direct effect of the normative feedback intervention on personal belief in anti-vaccine conspiracy theories immediately post-test was also significant, *b* = .19, *SE* = .08, *t*(160) = 2.43, *p* = .02, CI [.04, .35], however, this effect is increased when the mediator (change in perceived norms) is included, *b* = .32, *SE* = .08, *t*(161) = 3.97, *p* < .001, CI [.16, .48], indicating mediation. Confirming this, the indirect effect is significant, *b* = .13, *SE* = .05, 95% CI[.06, .25], showing that the normative feedback reduced perceived norms of other parents’ beliefs in anti-vaccine conspiracy theories which, in turn, reduced personal belief in anti-vaccine conspiracy theories.

### 3.7 Hypothesis 4: The mediating role of *perceived vaccination intention norm change* in the impact of the SNA intervention on vaccination intentions

**Fig 3:**
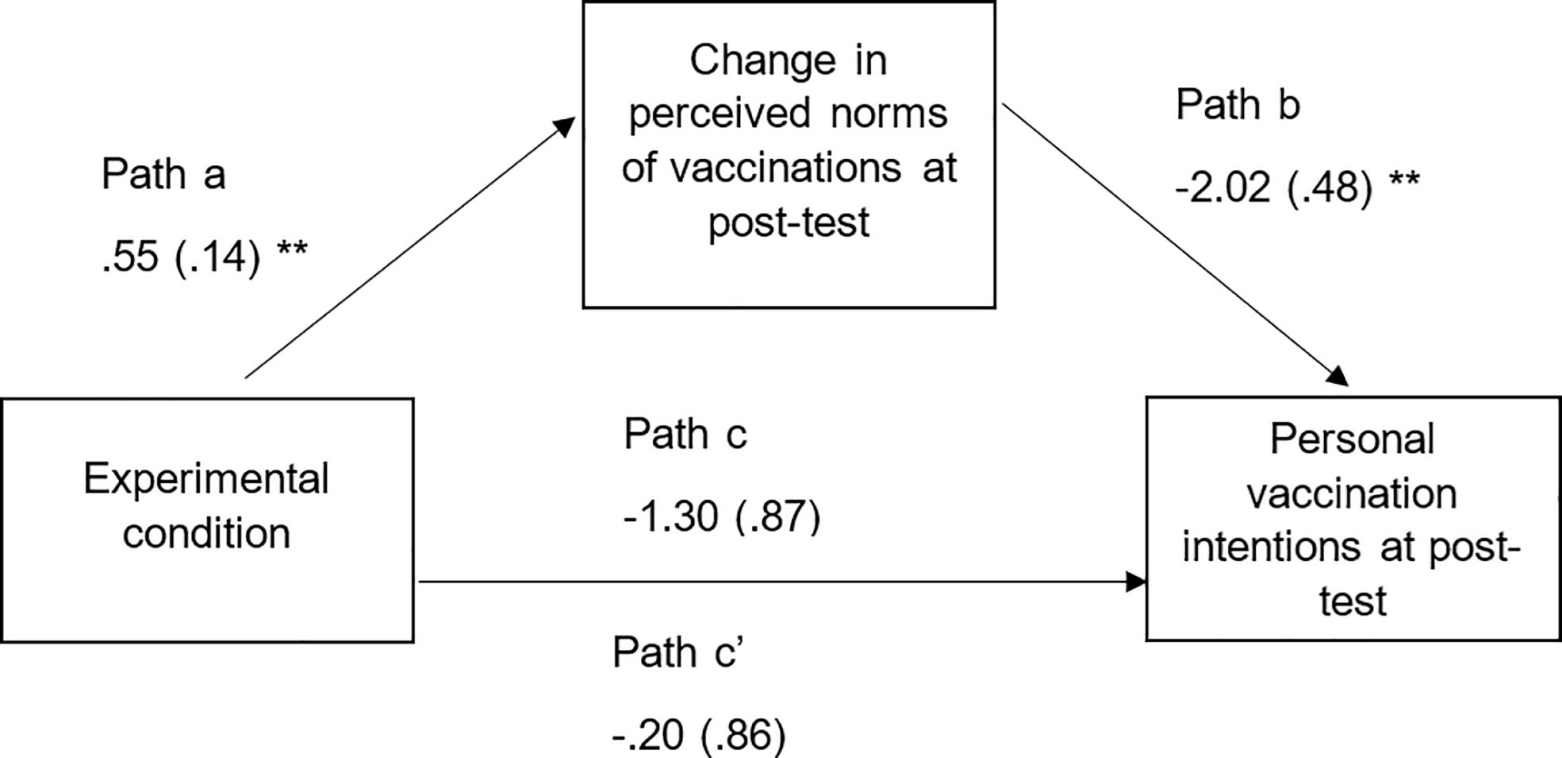
Model 4 showing indirect only mediation of the experimental condition on vaccination intentions through increased perceived norms of vaccination intentions. ** = p < .001.

Mediation analysis was employed again to test the hypothesis that the normative feedback increased perceived norms of other parents’ vaccination intentions from baseline to immediate post-test, which increased participants’ vaccination intentions. The change in perceived vaccination intentions variable was calculated by subtracting participants’ perceived vaccination intentions of other parents at immediate post-test from their baseline perceptions. Therefore, a negative number indicates that perceptions of other parents’ vaccination intentions have increased. The mediation analysis was conducted using Model 4 the PROCESS macro for SPSS, with 5000 bootstrapped samples [48]. Baseline vaccination intentions and baseline perceived norms of vaccination intentions were included in the model as covariates. The analysis supported the hypothesis (Fig 3). The normative feedback condition significantly predicted a change in perceived norms of other parents’ vaccination intentions at immediate post-test, *b* = .55, *SE* = .14, *t*(161) = 4.02, *p* < .001, 95% CI [.28, .81]. Similarly, a change in perceived vaccination intention norms at immediate post-test predicted personal vaccination intentions immediately after the intervention, *b* = -2.02, *SE* = .48, *t*(160) = -4.23, *p* < .001, 95% CI [-2.97, -1.08]. However, neither the direct effect *b* = -.20, *SE* = .86, *t*(160) = -.23, *p* = .82, 95% CI [-1.91, 1.51] or total effect *b* = -1.30, *SE* = .87, *t*(161) = -1.51, *p* = .13. 95% CI [-3.01, .41] of the normative feedback intervention on personal vaccination intentions immediately post-test were significant. The indirect effect of the normative feedback intervention on vaccination intentions was significant, *b* = -1.10, *SE* = .73, 95% CI [-2.84, -.02]. Meaning that the intervention increased perceived norms of vaccination intentions which in turn increasedpersonal vaccination intentions.

## 4. Discussion

The current research suggests that anti-vaccine conspiracy beliefs could be reduced via a brief normative feedback intervention based on the Social Norms Approach. Compared to an assessment only control, UK parents of young children who were exposed to the normative feedback intervention showed reduced belief in anti-vaccine conspiracy theories at immediate follow-up. Moreover, mediation analysis demonstrated the predicted mechanism; the intervention reduced perceived norms of anti-vaccine conspiracy beliefs, which in turn reduced personal beliefs. To our knowledge, our work is the first to showcase the possibility that normative feedback (as per the SNA) could be used as a technique to reduce anti-vaccine conspiracy beliefs. However, we also found that the effects of the intervention did not hold at the six-week follow-up, and there was no direct effect of the normative feedback on vaccination intentions. Mediation analysis however showed an indirect effect of the intervention on vaccination intentions; whereby the intervention increased perceptions of other parents’ vaccination intentions, which in turn increased personal vaccination intentions. Thus, further research exploring whether a top-up intervention could effectively maintain efficacy is warranted.

Our research has replicated and extended the work by Cookson et al., [6]. Specifically, we found that personal belief in anti-vaccine conspiracy theories was positively predicted by perceived norms of other parents’ belief in anti-vaccine conspiracy theories, and participants overestimated the extent to which other parents endorsed them. Building on Cookson, et al., [6], this study also demonstrated a similar pattern for vaccination intentions. Personal vaccination intentions were positively predicted by the perceived intentions of other UK parents, and participants under-estimated the vaccination intentions of other parents. Our present work consolidates the reasoning that both anti-vaccine conspiracy beliefs and vaccination intentions can be amendable via a SNA type intervention that challenges and reduces these misperceptions.

Furthermore, our findings showcased that the normative feedback intervention was successful in reducing anti-vaccine conspiracy beliefs at immediate post-test (partially supporting hypothesis 1). Participants who received normative feedback had a decreased belief in anti-vaccine conspiracy theories at the post-test measure. Our finding is important as this is the first time to our knowledge that a novel SNA type intervention has been used to reduce anti-vaccine conspiracy beliefs. Given the potential dangers of anti-vaccine conspiracy beliefs for health-protective behaviours (e.g., vaccine uptake), an intervention to address conspiracy beliefs has been long-awaited. However, the decrease in personal belief in anti-vaccine conspiracy theories did not hold for the six-week follow-up. At the six-week follow-up, participants’ conspiracy beliefs increased back to where they were at baseline. Previous research has used different lengths of time for a follow-up, and while four weeks had been considered short [17], as the SNA intervention tested here was brief, it was unlikely to remain effective for longer. Future research could investigate firstly, how long the effects of the intervention on reducing anti-vaccine conspiracy beliefs can hold, and secondly, whether a top-up intervention could be utilised to maintain changes in outcomes. For example, Neighbors et al. [49] concluded that a personalised normative feedback intervention to reduce drinking in heavy drinking college students was more successful when administered biannually as opposed to annually. A key strength of normative feedback interventions is the relative ease of disseminating the intervention and subsequent top-up feedback, which may be fruitful avenues for future SNA interventions focusing on reducing anti-vaccine conspiracy beliefs.

The current study also provided evidence for the mechanism through which the normative feedback is effective. Supporting hypothesis 3, mediation analysis demonstrated that the normative feedback reduced participants’ perceptions of other parents’ belief in anti-vaccine conspiracy beliefs, which in turn reduced their personal belief in anti-vaccine conspiracy theories. These findings support the focus on correcting misperceptions of anti-vaccine conspiracy beliefs and provide evidence that changing perceived norms directly influence anti-vaccine conspiracy beliefs.

However, the normative feedback did not directly increase vaccination intentions, and thus hypothesis 2 was not supported. One reason for this may be because the normative feedback was focused on correcting misperceptions of anti-vaccine conspiracy *beliefs* rather than correcting misperceptions of vaccination *intentions* (S1 File). Therefore, future research, which includes normative feedback that specifically compares participants’ vaccination intentions with their perceived norms of other parents’ vaccination intentions, and the ‘actual’ norm of parents’ vaccination intentions, may be successful. The lack of effect of the intervention in increasing vaccination intentions could also be due to a ceiling effect; participants’ baseline intentions to vaccinate were very high (*M* = 5.85 out of 7). Therefore, the participants in this study already had high vaccination intentions before the intervention, even though we attempted to recruit more hesitant participants about vaccines. Future research could focus vaccination interventions more specifically on participants who are hesitant about using vaccines. The analysis did however support hypothesis 4, where an indirect effect of the intervention on vaccination intentions was demonstrated. Therefore, this intervention does have the potential to correct misperceptions of vaccination intentions of other parents, which then in turn increases personal vaccination intentions.

### 4.1 Strengths and limitations

A limitation of the study lies in the way the feedback was presented to participants. In this study, participants in the normative feedback condition were given their normative feedback immediately after the baseline measures. The actual belief of other parents (which was compared to participants’ personal belief in anti-vaccine conspiracy theories at baseline) was taken from previous research by the authors [6], and the graphical element of the feedback (S1 File) did not include participants’ personal estimations of other parents’ beliefs in anti-vaccine conspiracy theories. The perceived norm was supplied in the feedback text only. Therefore, to further improve the intervention, the comparison of their own personal belief, their perceived norms and the actual norm could be more explicitly tailored to each participant. This could be important as if participants only paid full attention to the true norm presented in the graph, they could falsely construe their perceptions as accurate [50]. This is something that future research could incorporate, perhaps by taking baseline belief measures at a different time point. Similarly, as the experiment was delivered online, it is difficult to know how well the participants understood or attended to the normative feedback. Therefore, future research would benefit from qualitative approaches or a ‘think aloud’ [51] technique to help further refine this type of feedback to reduce belief in anti-vaccine conspiracy theories.

A further potential limitation of this study lies in the measurement of vaccination intentions used. The measure used in this study refers to a fictional disease ‘dysomeria’. Although this measure has been widely used in the literature (e.g., [25, 42, 46, 47]), it may not be viewed as threatening by participants as this disease is fictional. Therefore, responses to this measure may not align with vaccination intentions for known childhood diseases. To combat this limitation, future research could measure uptake intentions of actual childhood vaccines or longitudinal designs could measure actual vaccination behaviour, where ethical procedures would need to be carefully considered. It is also important to acknowledge that this study’s data collection was conducted during the COVID-19 pandemic. During this time, vaccination was an extremely topical issue, with them being described as the best chance to overcome the virus (e.g., [36]). Concurrently, anti-vaccine conspiracy beliefs associated with COVID-19 were emerging (e.g. [24]). Therefore, the backdrop of the pandemic could have influenced parents’ vaccination beliefs and intentions. However, baseline vaccination intentions of UK parents in this study (*M* = 5.85) were similar to those of UK parents measured in a previous study were data collection occurred in 2012 ([25], Study 1) (*M* = 5.63).

A key strength of this study is that it is the first, to our knowledge, to utilise an SNA type intervention, using normative feedback, to reduce UK parents’ beliefs in anti-vaccine conspiracy theories successfully. This is crucial as anti-vaccine conspiracy theories have been shown to lead to vaccine hesitancy, as demonstrated in this current study and previous research (e.g., Hornsey, Harris & Fielding, [27]; Jolley & Douglas, [25]). Vaccine hesitancy was listed as a top ten threat to global health in 2019 [29], and in the UK specifically, childhood vaccination rates are decreasing [7]. Moreover, during the current COVID-19 pandemic, addressing vaccine hesitancy could be vital to ensuring uptake of the COVID-19 vaccines [37].

Consequently, future research should focus on continuing to develop SNA type interventions to reduce anti-vaccine conspiracy beliefs and increase vaccination intentions. For example, this could involve ‘think aloud’ techniques, which involve participants talking aloud as they complete the intervention. Such an approach could be used to gain insights into how participants understand their feedback, particularly in vaccinations. Moreover, future research could focus on tailoring personalised feedback about participants’ perceptions of anti-vaccine conspiracy beliefs *and* vaccination intentions and gauging a better understanding of how a top-up intervention could improve the longevity of the effects. Finally, it should be acknowledged that the current study included only UK parents in the sample, and it is important that future research moves beyond using WEIRD samples.

Although further fine-tuning this type of intervention is warranted, the practical implications of this work are timely. Effective interventions to tackle anti-vaccine conspiracy beliefs and reduce vaccination intentions have long been called for (e.g. [42, 52]) but thus far have been limited. The SNA is one of the most widely used prevention approaches in the United States and is being used more globally [9]. The versatility of the approach and ease of application suggests that an online SNA using normative feedback has the potential to be applied as a practical strategy to attenuate anti-vaccine conspiracy beliefs and their consequences. Moroever, this type of intervention could be suggested for new or expectant parents as a pre-emptive approach.

### 4.2 Conclusion

In conclusion, this study has demonstrated that SNA normative feedback reduced perceptions of anti-vaccine conspiracy beliefs and increased perceptions of vaccination intentions of other parents, which in turn reduced personal anti-vaccine conspiracy beliefs and increased vaccination intentions. Our work demonstrates the utility of normative feedback to address anti-vaccine conspiracy beliefs in UK parents and is the first time, to our knowledge, that this technique has been used in this context. This research, therefore, provides the initial step in utilising normative feedback, where future research should focus on further understanding the use of this type of intervention to combat the dangers of conspiracy beliefs.

## Supporting information

S1 Table Analyses of variance of the effect of the intervention on personal beliefs in anti-vaccine conspiracy theories. (DOCX)

S2 Table Analyses of variance of the effect of the intervention on personal vaccination intentions. (DOCX)

S1 File Example of normative feedback. (DOCX)
