# Supplementary material for: A social norms approach intervention to address misperceptions of anti-vaccine conspiracy beliefs amongst UK parents
Source: PLoS One. 2021 Nov 12;16(11):e0258985. doi: 10.1371/journal.pone.0258985 (PMC8589151; doi:10.1371/journal.pone.0258985)
Supplement: S1 Table — (DOCX) [file pone.0258985.s001.docx]

**Table 3 Analyses of Variance of the effect of the intervention on personal beliefs in anti-vaccine conspiracy theories.**

|  | F(df) | P value | *η_p_^2^* |
| --- | --- | --- | --- |
| Time | .11 (2, 249.87) | .853 | .001 |
| Condition | .40 (1, 160) | .526 | .003 |
| Education | 2.92 (1, 160) | .089 | .02 |
| Time*Condition | 4.74* (2, 249.87) | .016 | .03 |
