# Supplementary material for: A social norms approach intervention to address misperceptions of anti-vaccine conspiracy beliefs amongst UK parents
Source: PLoS One. 2021 Nov 12;16(11):e0258985. doi: 10.1371/journal.pone.0258985 (PMC8589151; doi:10.1371/journal.pone.0258985)
Supplement: S2 Table — (DOCX) [file pone.0258985.s002.docx]

**Table 4 Analyses of Variance of the effect of the intervention on personal vaccination intentions.**

|  | F(df) | P value | *η_p_^2^* |
| --- | --- | --- | --- |
| Time | 2.39 (1.37, 222.57) | .113 | .015 |
| Condition | .09 (1, 162) | .767 | .001 |
| Education | 1.33 (1, 162) | .250 | .008 |
| Time*Condition | .55 (1.37, 222.57) | .515 | .003 |
