# Supplementary material for: A social norms approach intervention to address misperceptions of anti-vaccine conspiracy beliefs amongst UK parents
Source: PLoS One. 2021 Nov 12;16(11):e0258985. doi: 10.1371/journal.pone.0258985 (PMC8589151; doi:10.1371/journal.pone.0258985)
Supplement: S1 File — (DOCX) [file pone.0258985.s003.docx]

Example of Normative Feedback

**The development of vaccines is one of the most important advances in the history of medicine.**
Anti-vaccine conspiracy theories are conspiracy theories which attempt to discredit the effectiveness and safety of vaccines.
According to the information you provided us during the computer assessment, this is your level of belief in anti-vaccine conspiracy theories after viewing an article that promotes them ___
You also indicated how likely you would be to vaccinate a child against dysomeria. This was your answer, on a scale of 1 to 7, with 7 meaning that you would definitely vaccinate ___
You also completed questions asking what you believed other parents’ attitudes towards childhood vaccinations were. You told us that you believed the typical level of belief in anti-vaccine conspiracy theories of other parents was ___
You also estimated that they were this likely to vaccinate against dysomeria: **___**
Other parents’ belief in anti-vaccine conspiracy theories is actually very low, with average UK parents scoring 2 out of 7.

The graph below shows your belief, how much you predicted other British parents believed in anti-vaccine conspiracy theories and the actual belief of other parents in anti-vaccine conspiracy theories:


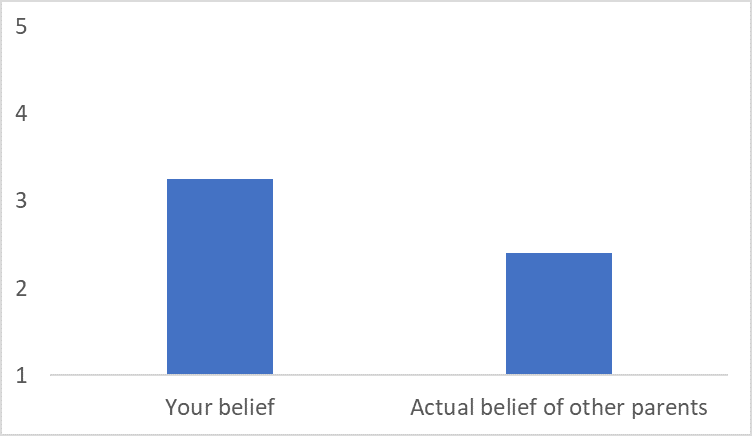


We significantly over-estimate how much other parents believe in these conspiracy theories and therefore have a distorted view of how common they are. Parents’ actual belief in anti-vaccine conspiracy theories is much lower than people think it is.

**The overwhelming majority of UK parents choose to vaccine their children.**

**Overall, over 90% believe that childhood vaccines are fully safe and effective.**
